# 

**Published:** 1854-01

**Authors:** 


### THE MISSISSIPPI VALLEY ASSOCIATION OF DENTAL SURGEONS.

It will be recollected by the members of this association, that the time for holding its annual meeting has been changed to the third Wednesday of February. The society will meet at 10, A. M., of Wednesday, 22d of February next, at the Ohio College of Dental Surgery. We hope to see a full attendance.

---

### OHIO DENTAL COLLEGE ASSOCIATION.

The annual meeting of this association takes place this year on Monday, 10 A. M., 20th of February next, at the Ohio College of Dental Surgery. The commencement exercises of the College comes off on Wednesday evening. It is hoped that the stockholders will generally attend, and we shall be happy to see as many of the Profession present as can attend.

---

### PAYMENTS FOR THE REGISTER.

In each number of the Register we receipt the amount paid in by each subscriber during the quarter. By reference to this table, subscribers can see how their account stands. If any mistake is made in this table we hope to be informed of it, and it will be corrected. Remittances by mail will be at our risk. Those who find their names not in this list during the year, will please recollect that the printer *must be paid.*

### RECEIPTS FOR THE REGISTER.

Dr. A. Berry, Covington, Ky., Vol. 7, $2,00

Dr. E. L. Holmes, Lockport, N. Y., vol. 7,........ 2,00

Dr. M. Kelley, Bentonville, Ia. vol. 7, 2,00

Dr. D. Watters, Center P. O., Montgomery co., O., vols. 4, 5 and 6,.. 6,00

Dr. J. A. Kennicott, Chicago, Ill., vol. 4, 5 and 6,........ 6,00

Dr. Johnson, Indianapolis, Ia. vol. 7 2,00

Dr. L. Claggett, Georget'n, Ky. vol. 6, 2,00

Dr. M. L. Pierce, Madina P. O., Olean co., N. Y., vol. 7........ 2,00

Dr. J. Taft, Xenia, O., vol. 6 and 7,... 4.00

Dr. G. Watt, Xenia, O., vol. 6 and 7,. 4,00

Dr. E. I. Poof, Fort Madison, Iowa, vol. 7,........ 2,00

Dr. B. A. Satterthwaite, Lima, O., vol. 7 2,00

### Dental Journals Received.

AMERICAN JOURNAL OF DENTAL SCIENCE, for October.
DENTAL NEWS LETTER " "
DENTAL RECORDER " "
DENTAL EXPOSITOR " May.

### Medical Journals Received.

THE SOUTHERN JOURNAL OF THE MEDICAL AND PHYSICAL SCIENCES.
THE ST. LOUIS MEDICAL AND SURGICAL JOURNAL.
BOSTON MEDICAL AND SURGICAL JOURNAL.
CHARLESTON MEDICAL AND SURGICAL JOURNAL.
THE MEDICAL CHRONICLE, or MONTREAL MONTHLY JOURNAL OF MEDICINE AND SURGERY.
OHIO MEDICAL JOURNAL.
WESTERN LANCET.
IOWA MEDICAL JOURNAL.

### Baltimore College of Dental Surgery.

### Fourteenth Session, 1853–'54.

CHAPIN A. HARRIS, A. M., M. D.,
Professor of Principles and Practice of Dental Surgery.

THOMAS E. BOND, Jr., A. M., M. D.,
Professor of Special Pathology and Therapeutics.

WASHINGTON R. HANDY, M. D.,
Professor of Anatomy and Physiology.

ALFRED A. BLANDY, M. D.,
Professor of Operative Dentistry.

PHILIP H. AUSTEN, A. M., M. D.,
Professor of Mechanical Dentistry.

REGINALD N. WRIGHT, A. M., M D.,
Lecturer on Chemistry and Metallurgy.

The regular course of Lectures will commence on the 1st of November and close the 1st of March. The Infirmary, Mechanical and dissecting rooms will be open on the 1st Monday in October, during which month there will be occasional lectures from each of the Professors.

Tickets for the Course, $110. Dissecting Ticket, (optional), $10. Diploma Fee, $30. Matriculation Fee, $5.

P. H. AUSTEN, Dean,
84 *Sharp Street, Baltimore.*

---

NINTH ANNUAL ANNOUNCEMENT
OF

### THE OHIO COLLEGE OF DENTAL SURGERY.

### Session of 1853–'54.

The regular course of lectures in this institution, commences the first Monday of November, and closes on the third Wednesday of February.

**FACULTY.**

JAMES TAYLOR, M. D., D. D. S.
*Professor of the Principles and Practice of Dental Surgery.*

THOMAS WOOD, M. D.,
*Professor of Anatomy and Physiology.*

J. B. SMITH, M. D.,
*Professor of Pathology and Therapeutics.*

JOHN ALLEN, D. D. S.,
*Professor of Operative Dentistry.*

H. R. SMITH, D. D. S.
*Professor of Mechanical Dentistry.*

G. WATT, M. D.,
*Lecturer on Chemistry,*

Tickets for the course, $100,00 ; Matriculating Ticket, 5,00 ; Diploma Fee, $25,00. Good board can be had at from $2,50 to $3,00 per week. JAMES TAYLOR, *Dean.*

### A Card to the Dental Profession.

The undersigned would respectfully inform the members of the Dental Profession, that they have perfected a plan of mounting teeth on Atmospheric plates, which entirely overcomes all the difficulties heretofore, to wit: the warping of plates and a variety of other difficulties. For which they have obtained Letters Patent, and will furnish office rights on such reasonable terms, that we think no operator will mount a set of teeth without it, after knowing its advantages over the old method.

Those residing in the following states and territories may be supplied with office rights by J, K. Rickey, Keokuk, Iowa, to wit.; New York, Pennsylvania, Indiana, Missouri, Illinois, Michigan, Wisconsin, Louisiana, Texas, California, Maine, Vermont, Rhode Island, South Carolina, Iowa, Florida, Minnesota, and Oregan Territories. In the remaining Territories, by Creder & Williams of Lancaster, Ohio.

J. K. RICKEY, Keokuk, Iowa.
H. L. CRIDER & D. WILLIAMS, Lancaster, Ohio.

---

### A Card to the Profession.

I have recently received letters, giving information that there are those going about, claiming to be my agents for vending my improved method of mounting teeth with continuous gums, who are unauthorized by me. I would therefore inform the Profession, that my agents will be furnished with the necessary documents from me to make their contracts valid ;and agents should be required, in all cases where negotiations are being made, to exhibit their credentials. I wish to avoid litigation in all cases where it is possible, although it has become necessary, in a few instances, to institute suits for infringements. This has been rendered necessary with Dr. Hunter, as well as a suit for libel.

Having found it necessary to take out letters patent to secure the benefit of my improvement, I shall most assuredly defend this right, that those who purchase may be protected in its use.

Jan. 1, 1851. J. ALLEN.

---

### Removal.

W. Z. REES Respectfully informs his friends and patrons, that he has REMOVED his Manufactory to Sixth Street, between Walnut and Vine, South side. A general assortment of DENTAL and SURGICAL INSTRUMENTS always on hand and made to order. Also a general assortment of Pearl Handles and Mirrors always on hand

